# Supplementary figures and images for: AlphaFold2-guided engineering of split-GFP technology enables labeling of endogenous tubulins across species while preserving function
Source: PLoS Biol. 2024 Aug 19;22(8):e3002615. doi: 10.1371/journal.pbio.3002615 (PMC11361732; doi:10.1371/journal.pbio.3002615)

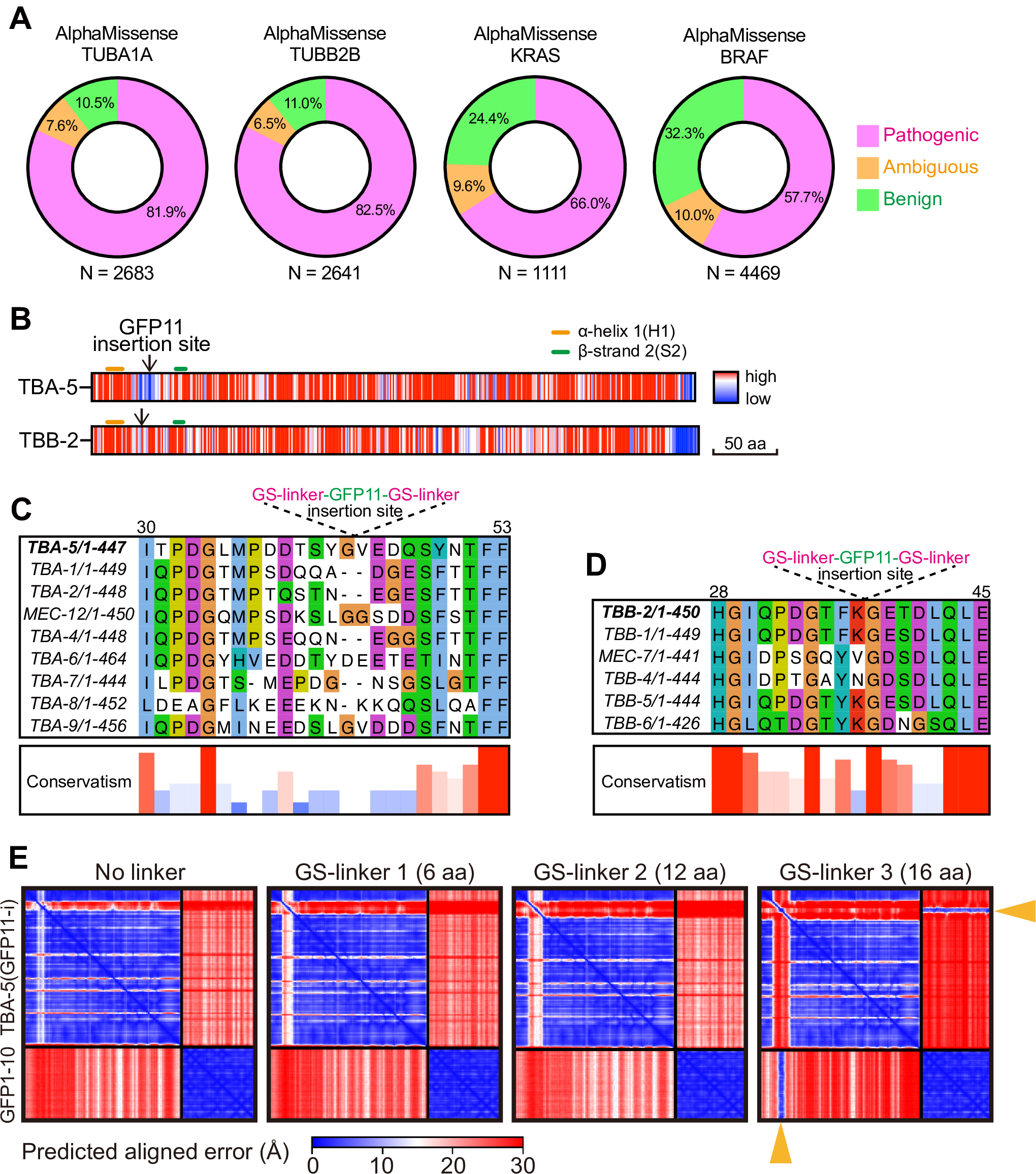

Supplement: S1 Fig — (A) AlphaMissense-based impact predictions of missense mutations in human α-tubulin TUBA1A, β-tubulin TUBB2B, KRAS, or BRAF proteins. The impact of mutations are categorized into 3 distinct groups: likely pathogenic (magenta), ambiguous (orange), and likely benign (green). N indicated the number of predicted mutation cases by AlphaMissense. (B) Conservatism of all amino acids along TBA-5 and TBB-2 sequences. Conservatism ranged from low (blue) to high (red). Positions of H1 and S2 were indicated above each sequence. GFP11-i insertion sites were indicated by black arrows. (C) Sequence alignment of all 9 C. elegans α-tubulins at H1-S2 loops. The numbers above sequences indicated residue positions in TBA-5. GFP11 flanked by GS-linker was inserted between Gly43 and Val44 in TBA-5. (D) Sequence alignment of all 6 C. elegans β-tubulins at H1-S2 loops. The numbers above sequences indicated residue positions in TBB-2. GFP11 flanked by GS-linker was inserted between Lys37 and Gly38 in TBB-2. (E) Representative PAE plots for each prediction in Fig 1A. Plots were generated by AlphaFold2 and expected position error (Å) ranged from 0 (blue) to 30 (red). Positions of GFP11 in TBA-5 (GFP11-i) were indicated by orange arrowheads. GFP, green fluorescent protein; PAE, predicted alignment error. (TIF) [file pbio.3002615.s001.tif]

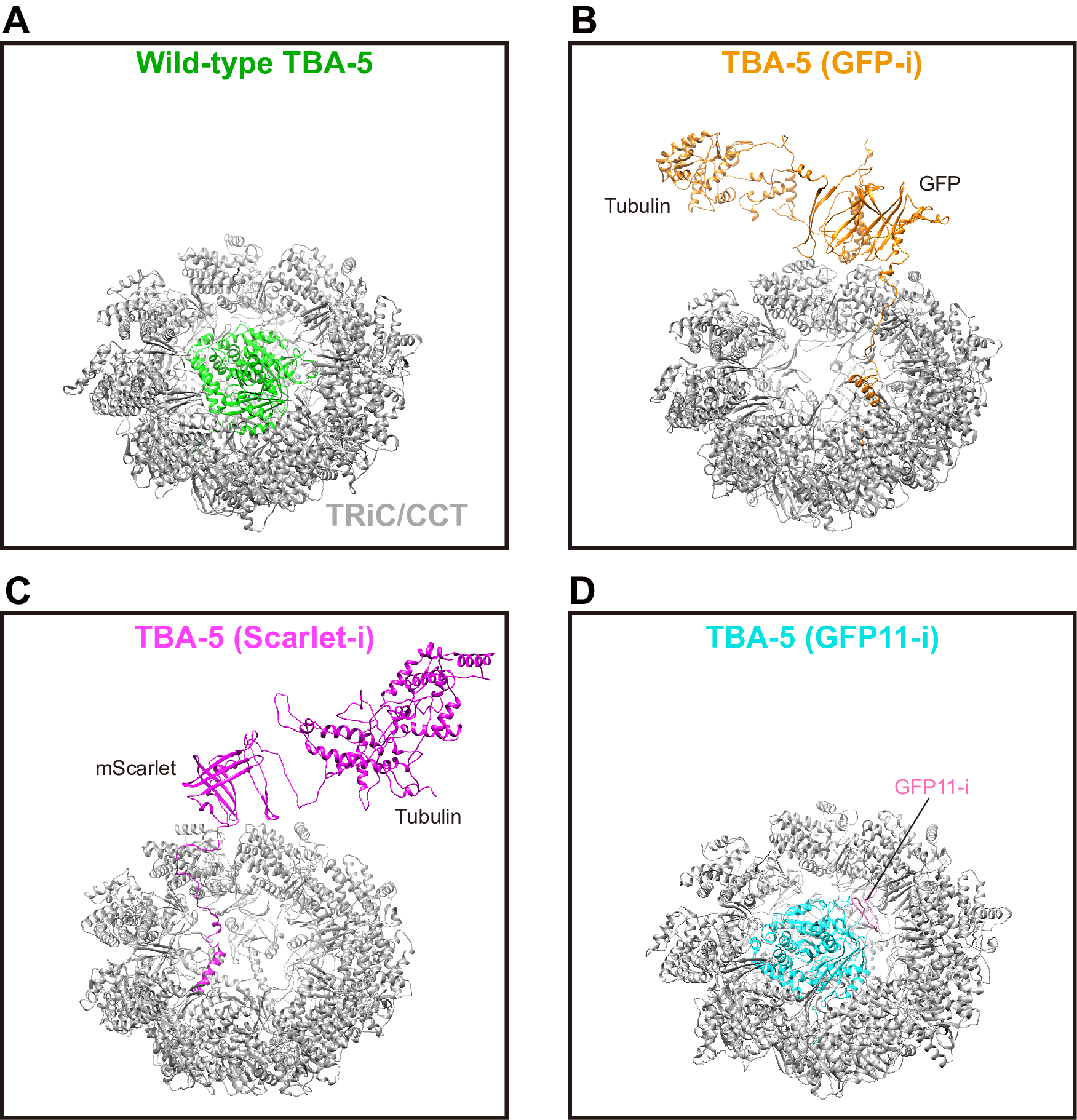

Supplement: S2 Fig — (A) Structural model of untagged (wild-type) α-tubulin TBA-5 (green) with the TRiC / CCT chaperone complex after Rosetta relax runs. Wild-type CeTBA-5 was employed to substitute the template tubulin in 7TUB model (RCSB PDB database) for relax runs. Initially, substituted tubulin was deposited inside the TRiC / CCT complex, and TRiC / CCT complex was set to be rigid. (B) Structural model of internal GFP-tagged α-tubulin TBA-5 (orange) with the TRiC / CCT chaperone complex after Rosetta relax runs. Full-length GFP was inserted into the H1-S2 loop of TBA-5. Then, GFP-tagged TBA-5 was employed to substitute the template tubulin in 7TUB for relax runs. (C) Structural model of internal mScarlet-tagged α-tubulin TBA-5 (magenta) with the TRiC / CCT chaperone complex after Rosetta relax runs. Full-length mScarlet was inserted into the H1-S2 loop of TBA-5. Then, mScarlet-tagged TBA-5 was employed to substitute the template tubulin in 7TUB for relax runs. (D) Structural model of GFP11-i -tagged α-tubulin TBA-5 (cyan) with the TRiC / CCT chaperone complex after Rosetta relax runs. GFP11-i (GFP11 flanked by GS-linker 3) (colored in pink) was inserted into the H1-S2 loop of TBA-5. Then, TBA-5 (GFP11-i) was employed to substitute the template tubulin in 7TUB for relax runs. (TIF) [file pbio.3002615.s002.tif]

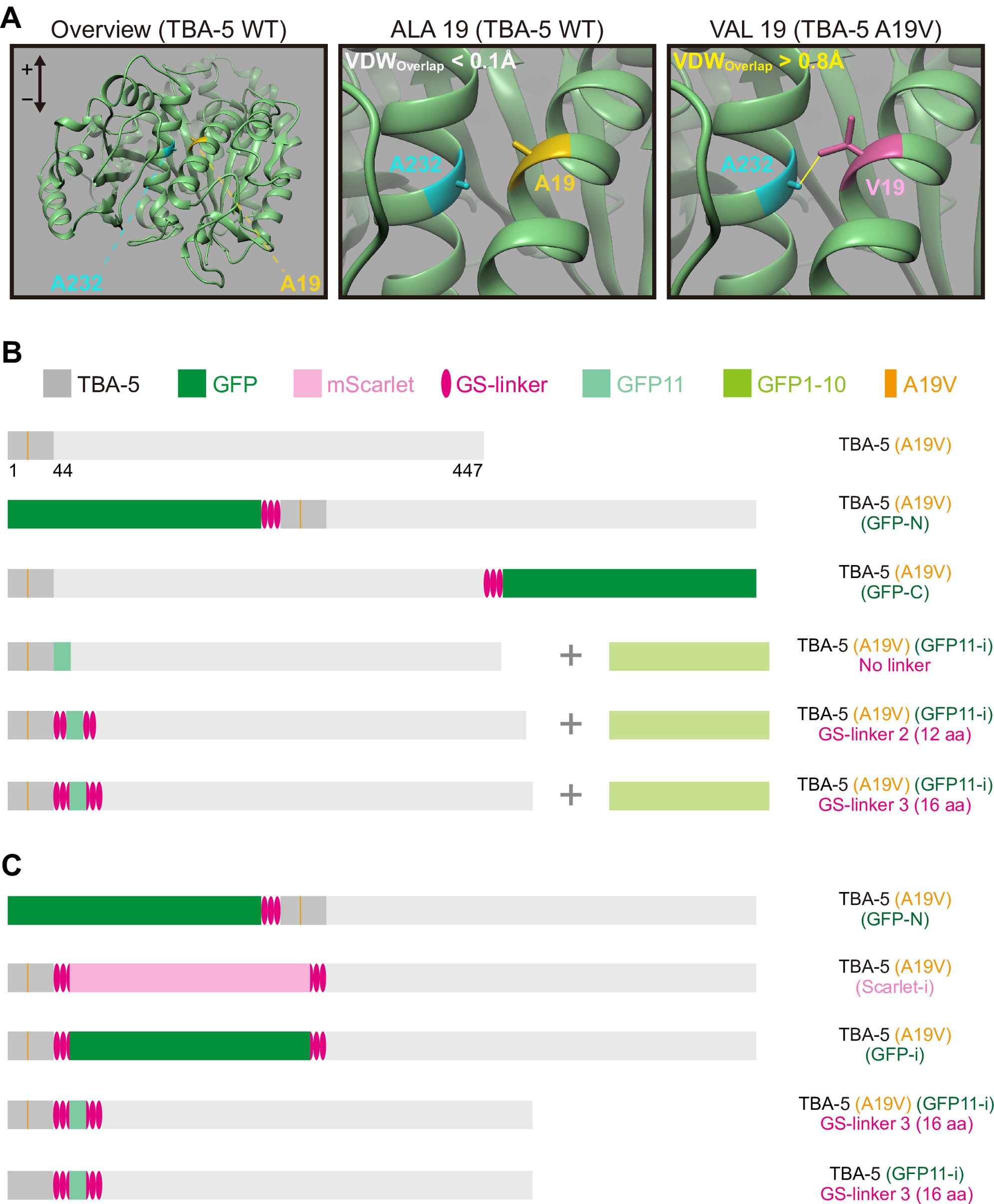

Supplement: S3 Fig — (A) Structural illumination of wild-type TBA-5 and TBA-5 (A19V). Ala19 was indicated in yellow, Val19 was indicated in magenta, and Ala232 was indicated in cyan. VDWOverlap represented the overlapped distance of van der Waals radius between protruding carbon in A232 and proximal carbon in A19 or V19, which was calculated using ChimeraX software (see also Materials and methods). Clashed atoms were connected with yellow lines. “+” indicated plus end when TBA-5 was assembled into MTs. (B) Linear representation of distinctly labeled TBA-5 (A19V) constructs for OE. GFP-N, gfp::3xgs-linker::tba-5; GFP-C, tba-5::3xgs-linker::gfp; GFP11-i, tba-5(1–43)::gs-linker::gfp11::gs-linker::tba-5(44–447). The length of one unit of GS linker in magenta was 6 aa. (C) Linear representation of fluorescent labeled TBA-5 (A19V) constructs for KI. Scarlet-i, tba-5(1–43)::gs-linker 3::mscarlet::gs-linker 3::tba-5(44–447); GFP-i, tba-5(1–43)::gs-linker 3::gfp::gs-linker 3::tba-5(44–447). KI, knock-in; MT, microtubule; OE, overexpression. (TIF) [file pbio.3002615.s003.tif]

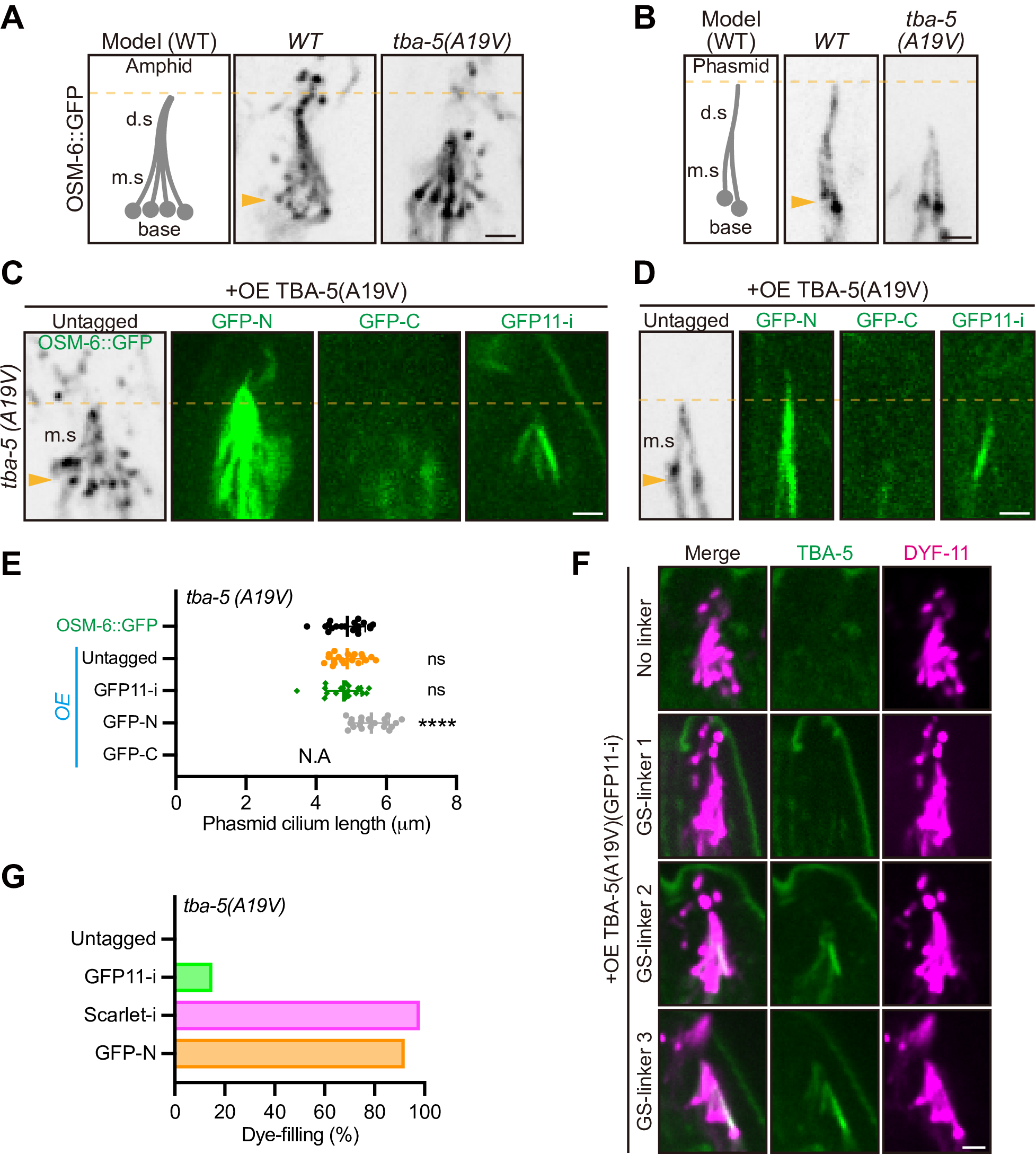

Supplement: S4 Fig — (A and B) Morphologies and models of amphid (A) and phasmid (B) cilia in wild-type or tba-5 (A19V) animals at 15°C. Cilia were visualized using endogenous OSM-6::GFP. Ciliary bases were indicated by orange arrowheads. Scale bar, 2 μm. (C and D) Morphologies of amphid (C) and phasmid (D) cilia in tba-5 (A19V) animals with transgenic expression of tba-5 (A19V) variants. “Untagged” indicated transgenic expression of untagged TBA-5 (A19V). Cilia were visualized using OSM-6::GFP (for untagged group) or labeled TBA-5 (A19V). The detailed GFP-N, GFP-C or GFP11-i constructs could be found in S3B Fig. All independent OE lines (≥3 lines) in each group exhibited similar phenotype. Ciliary bases were indicated by orange arrowheads. Scale bar, 2 μm. (E) Phasmid cilium length in different tba-5 (A19V) transgenic lines. Cilia in untagged tba-5 (A19V) animals were visualized using OSM-6::GFP. Cilia in other transgenic lines were visualized using labeled TBA-5 (A19V). N = 20 for each group. (F) Phenotypes of amphid cilia in tba-5 (A19V) animals with transgenic expression of TBA-5 (A19V) (GFP11-i). gfp11-i constructs without linker, or flanked by GS-linker 1, or GS-linker 2, or GS-linker 3, were overexpressed with gfp1-10 constructs in tba-5 (A19V) animals. The detailed GFP11-i constructs could be found in S3B Fig. Cilia were visualized using endogenous DYF-11::wrmScarlet. All independent lines (≥3 lines) in each group exhibited similar phenotype. Scale bar, 2 μm. (G) Percentage of dye-filling positive amphids when endogenous TBA-5 (A19V) were labeled using different tags. N = 100 for each group. Numerical data for panels E and G are available in S1 Data. d.s., distal segment; m.s., middle segment; N.A, not applicable; OE, overexpression. (TIF) [file pbio.3002615.s004.tif]

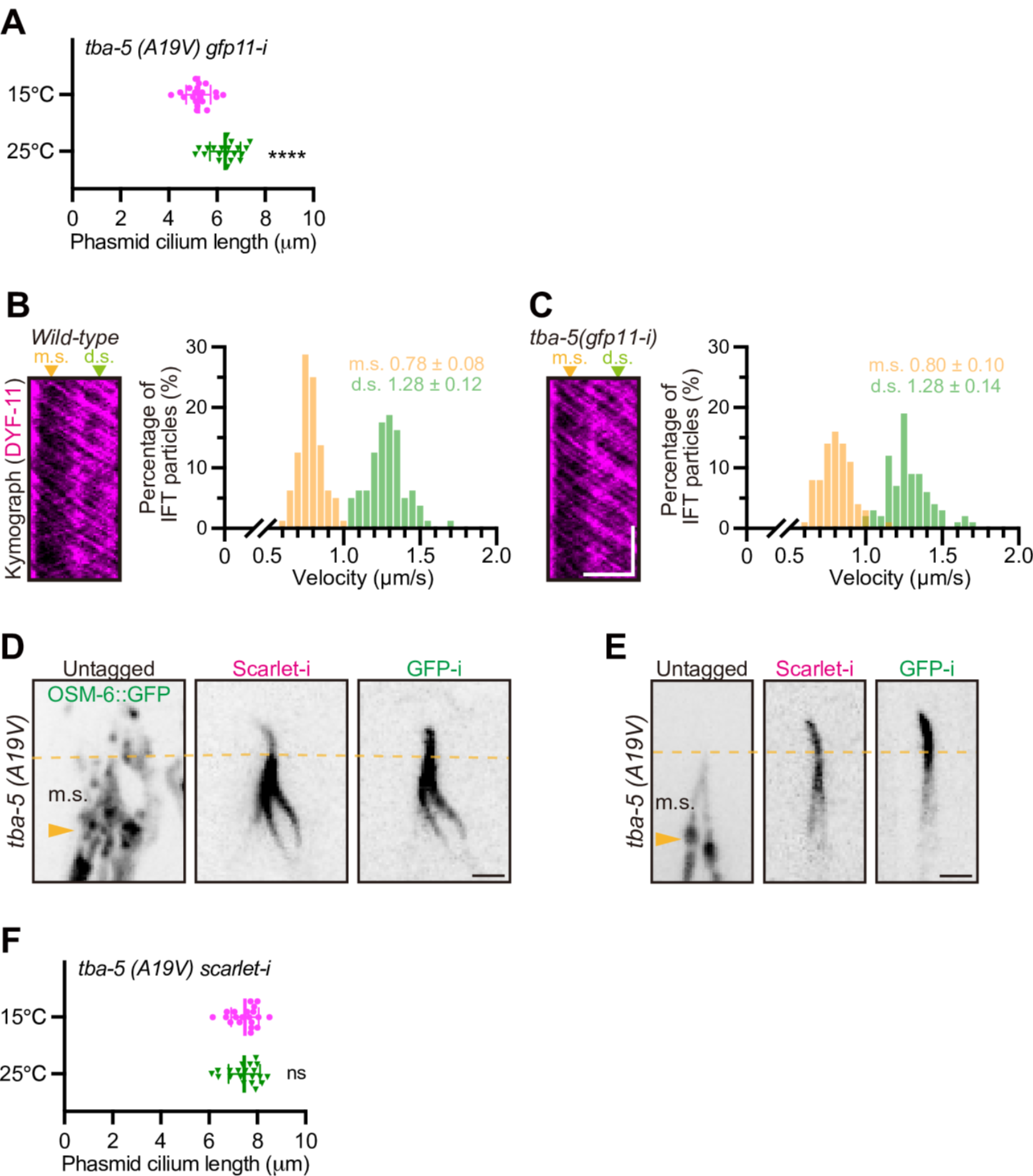

Supplement: S5 Fig — (A) Phasmid cilium length in tba-5 (A19V) (gfp11-i) animals at 15 or 25°C. N = 20 for each group. (B and C) IFT velocities in cilia of wild-type (B) or tba-5 (gfp11-i) KI (C) animals. Representative kymographs of IFT were shown on the left (DYF-11::wrmScarlet as marker); histograms of IFT velocities were shown on the right. Data were show as mean ± SD; N = 80 for each group. Scale bar, 2 μm and 5 s. (D and E) Morphologies of amphid cilia (B) and phasmid cilia (C) when endogenous TBA-5 (A19V) were labeled with internal mScarlet (Scarlet-i) or GFP (GFP-i). OSM-6::GFP marked the defective cilia when TBA-5 (A19V) was untagged. Scale bar, 2 μm. (F) Phasmid cilium length in tba-5 (A19V) (scarlet-i) KI animals at 15 or 25°C. N = 20 for each group. Numerical data for panels A, B, C, and F are available in S1 Data. d.s., distal segment; IFT, intraflagellar transport; KI, knock-in; m.s., middle segment. (TIF) [file pbio.3002615.s005.tif]

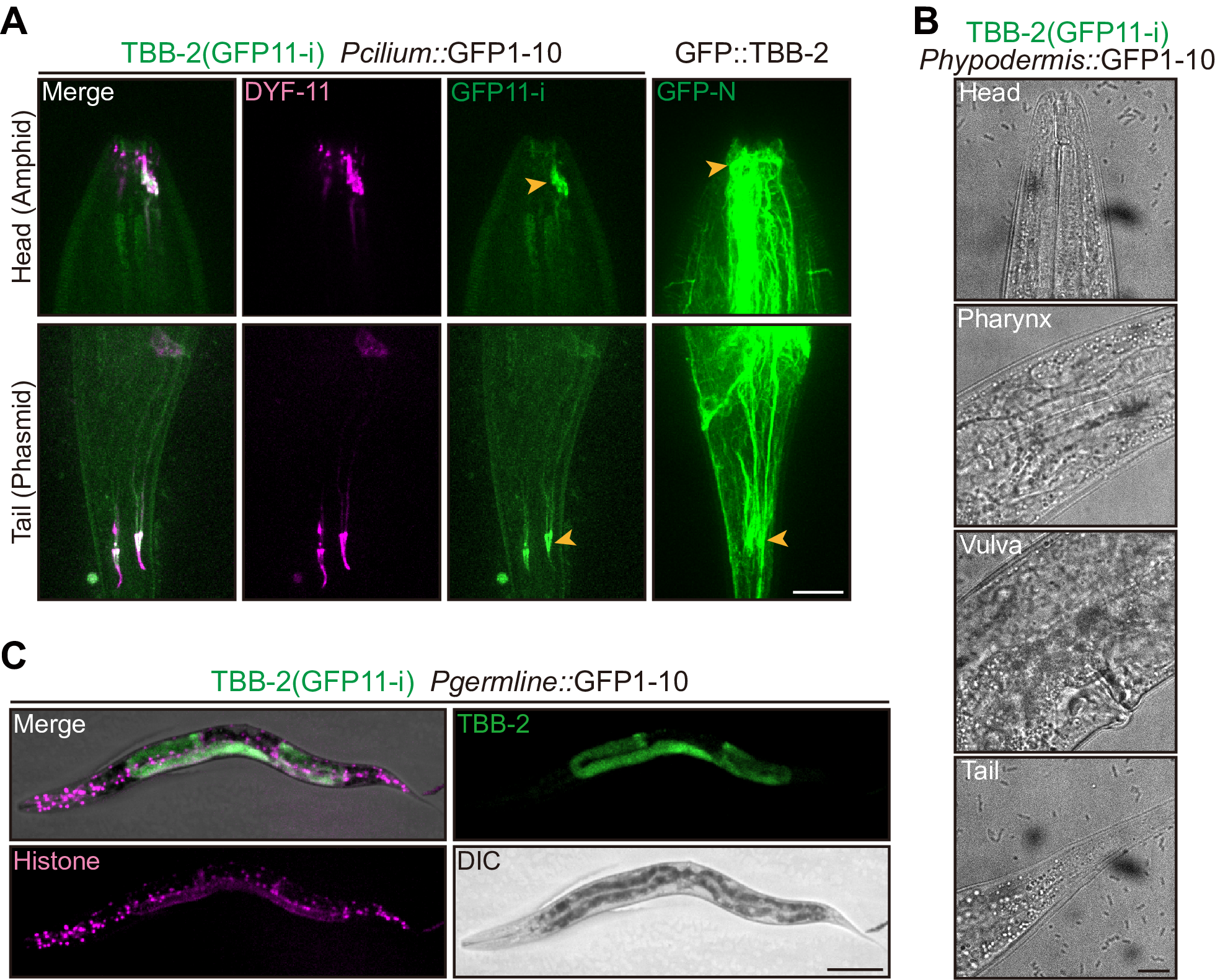

Supplement: S6 Fig — (A) Representative images of endogenous TBB-2 (GFP11-i) with ciliated neuron-specific GFP1-10, or endogenous GFP::TBB-2, in C. elegans heads and tails. Fluorescence of TBB-2 (GFP11-i) images were enhanced 6 times when compared to GFP::TBB-2 images. Supposed positions of amphid and phasmid cilia were indicated by orange arrowheads. DYF-11::wrmScarlet marked the sensory cilia. Scale bar, 10 μm. (B) DIC (bright field) images corresponding to the head, pharynx, vulva, and tail in Fig 4C. Scale bar, 10 μm. (C) Representative images of endogenous TBB-2 (GFP11-i) with germline-specific GFP1-10, along the whole body of young-adult worm. Scale bar, 100 μm. DIC, differential interference contrast; GFP, green fluorescent protein. (TIF) [file pbio.3002615.s006.tif]

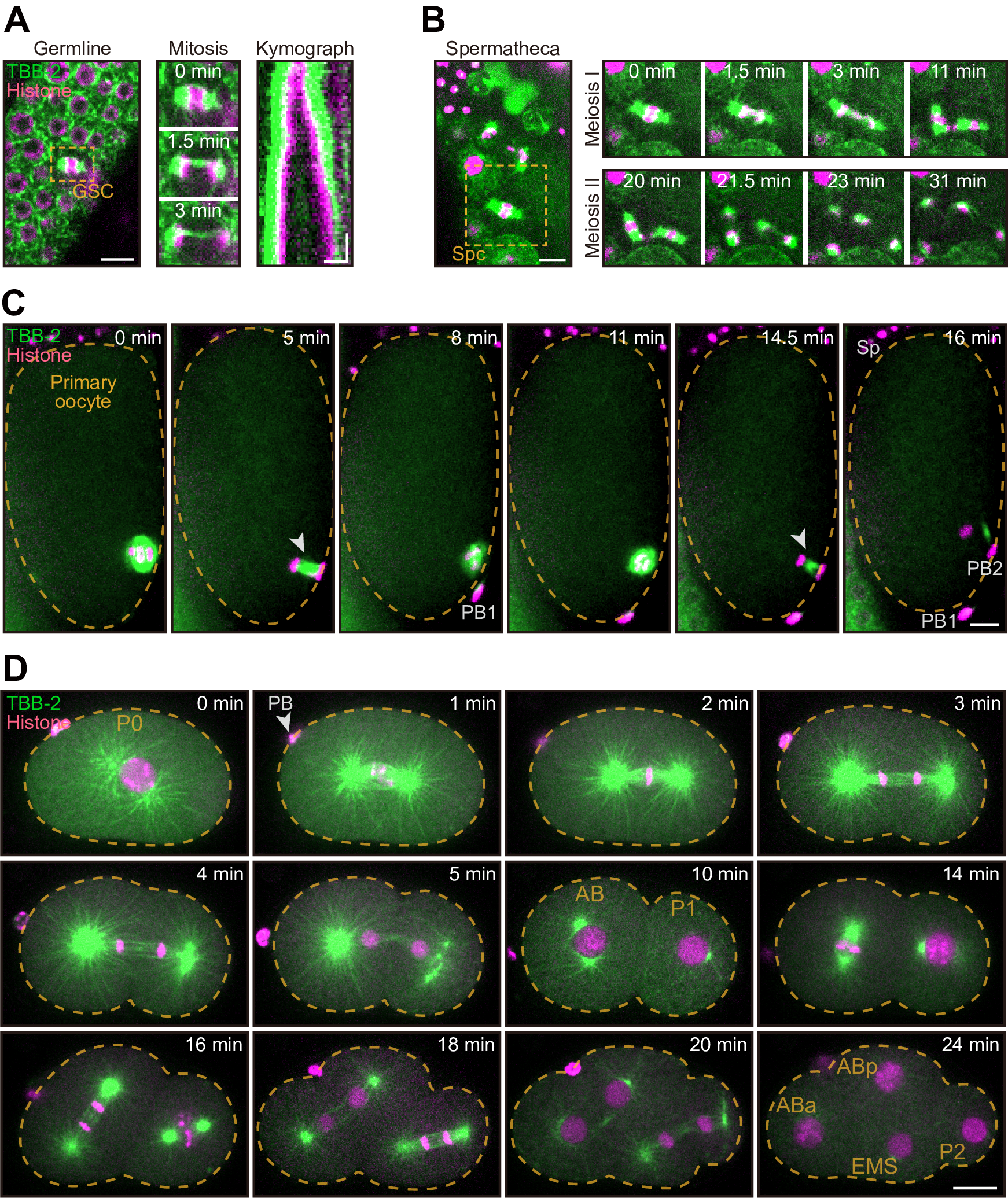

Supplement: S7 Fig — (A) (Left) Mitosis of a GSC. Scale bar, 5 μm. (Middle) Stills from mitosis of GSC indicated within orange dashed box (see also S6 Video). (Right) Kymograph of mitosis of GSC indicated with orange dashed box. Scale bar (horizontal), 2 μm; scale bar (vertical), 2 s. (B) (Left) Meiosis of a primary spermatocyte (Spc). Scale bar, 5 μm. (Right) Stills from meiosis of primary spermatocyte indicated with orange dashed box (see also S7 Video). This included meiosis I and meiosis II. (C) Stills from meiosis of a fertilized oocyte in utero (see also S8 Video). This included meiosis I and meiosis II. Spindles in anaphase I and anaphase II were indicated by white arrowheads. Edges of cells were depicted by orange dashed curves. PB1, the first polar body; PB2, the second polar body; Sp, sperm. Scale bar, 5 μm. (D) Stills from embryonic cleavage of a single-cell zygote (P0) (see also S9 Video). “0 min” represented the time just before nuclear envelope breakdown. Edges of cells were depicted by orange dashed curves. AB and P1 were daughter cells in 2-cell embryos. ABa, ABp, EMS, and P2 were daughter cells in 4-cell embryos. Scale bar, 10 μm. GSC, germline stem cell; MT, microtubule; PB, polar body. (TIF) [file pbio.3002615.s007.tif]

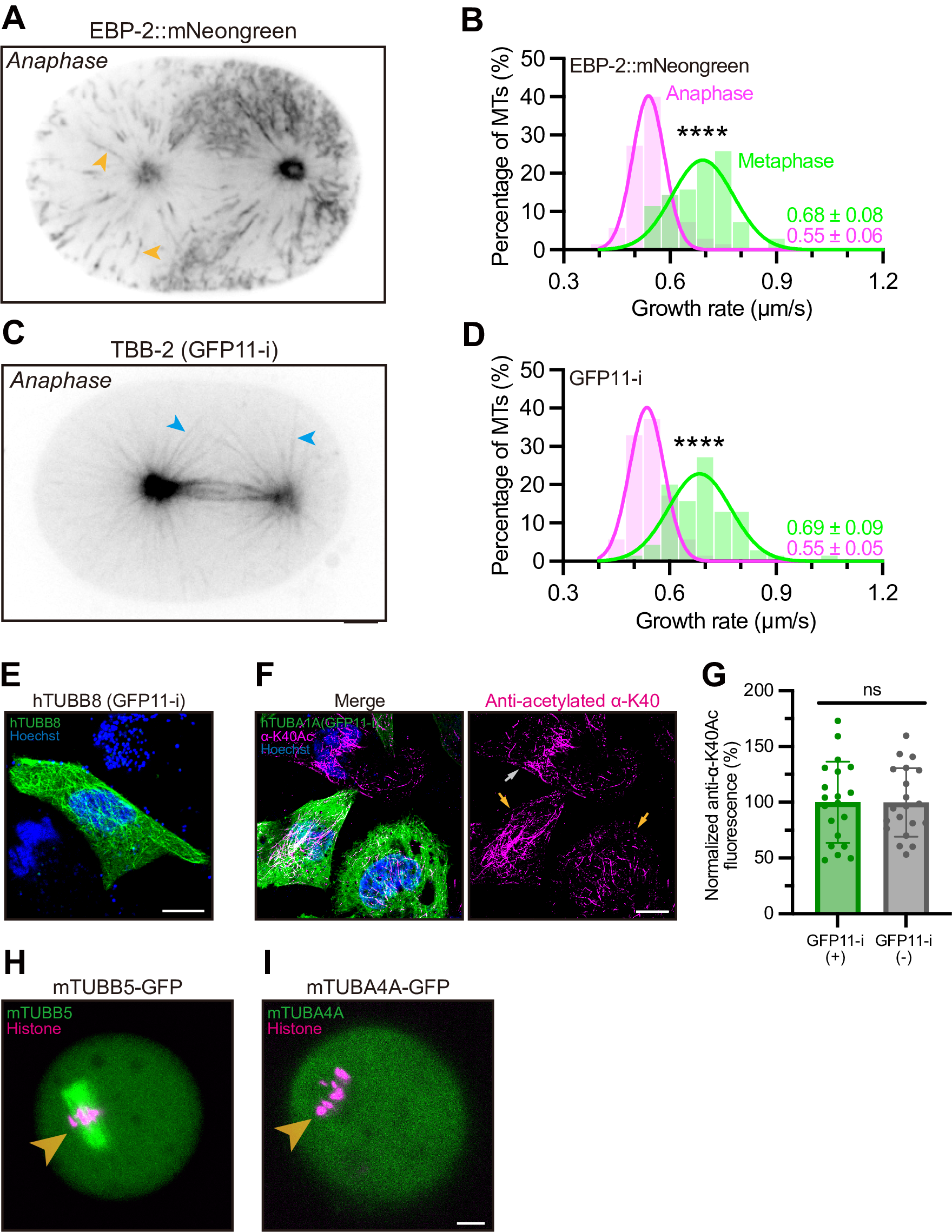

Supplement: S8 Fig — (A) Representative image of single-cell embryo in anaphase using EBP-2::mNeongreen as markers. A total of 10 frames (400 ms exp.) were projected as a single image to show the paths of EBP-2 dots, as indicated by orange arrowheads (which represented the subset of growing astral MTs). (B) Histogram showing the distribution of astral MT growth rates in metaphase and anaphase using EBP-2::mNeongreen as markers. Green lines (metaphase) and magenta lines (anaphase) showed Gaussian fit curves. Quantitative data were shown as mean ± SD. N = 70 MTs for each group. (C) Representative image of single-cell embryo in anaphase using TBB-2 (GFP11-i) as markers. A total of 10 frames were projected as a single image. Some astral MTs were indicated by blue arrowheads. Scale bar, 5 μm. (D) Histogram showing the distribution of astral MT growth rates in metaphase and anaphase using TBB-2 (GFP11-i) as markers. N = 70 MTs for each group. (E) Representative images for transfection-positive cells when transfected with htubb8 (gfp11-i) (100 ng) and gfp1-10 (200 ng). Scale bar, 10 μm. (F) Representative image for immunostaining of acetylated α-tubulin K40 residue (α-K40Ac, magenta) in transfection-positive cells (indicated by orange arrowheads) and transfection-negative cells (gray arrowhead). Scale bar, 10 μm. (G) Whole-cell average anti-αK40Ac fluorescence in transfection-positive cells (+) and transfection-negative cells (−). N = 20. (H) Representative images for mouse oocytes in metaphase I showing localization of mTUBB5-GFP and histone. Meiotic spindles were indicated by orange arrowheads. (I) Representative images for mouse oocytes in metaphase I showing localization of mTUBA4A and histone. Meiotic spindles were indicated by orange arrowheads. Scale bar, 10 μm. Numerical data for panels B, D, and G are available in S1 Data. GFP, green fluorescent protein; MT, microtubule. (TIF) [file pbio.3002615.s008.tif]
