# Supplementary material for: AlphaFold2-guided engineering of split-GFP technology enables labeling of endogenous tubulins across species while preserving function
Source: PLoS Biol. 2024 Aug 19;22(8):e3002615. doi: 10.1371/journal.pbio.3002615 (PMC11361732; doi:10.1371/journal.pbio.3002615)
Supplement: S1 Table — (DOCX) [file pbio.3002615.s018.docx]

**S1 Table.** *C. elegans* strains in this study.

| **Strain Name** | **Genotype** | **Source** |
| --- | --- | --- |
| N2 | *Wild-Type* | N.A. |
| SP2101 | *mnIs17 [osm-6::GFP unc-36(+)]* | N.A |
| JIM113 | *ujls113*  *[Ppie-1::H2B::mCherry; Pnhr-2::his-24::mCherry II]* | N.A |
| PHX7618 | *tbb-2 (gfp11-i) (syb7618)* | SunyBiotech. |
| PHX4515 | *che-3::t2a::sfgfp1-10 (syb4515)* | SunyBiotech. |
| SHX2025 | *Pcol-19::sfgfp1-10 single copy KI (zjuSi177)* | Zhejiang Univ. |
| DUP228 | *glh-1::t2a::sfgfp1-10 (sam129)* | Zhejiang Univ. |
| GOU4772 | *dyf-11::wrmScarlet (cas1086); him-5 (e1490)* | Microinjection |
| GOU5351 | *tbb-2 (gfp11-i) (syb7618); cas1086;*  *che-3::t2a::sfgfp1-10 (syb4515)* | Genetic cross |
| GOU5352 | *tbb-2 (gfp11-i) (syb7618); Pcol-19::sfgfp1-10 (zjuSi177)* | Genetic cross |
| GOU5353 | *tbb-2 (gfp11-i) (syb7618);*  *glh-1::t2a::sfgfp1-10 (sam129)* | Genetic cross |
| GOU5354 | *gfp::tbb-2 (cas970)* | Microinjection |
| GOU5355 | *tbb-2::gfp (cas722)* | Microinjection |
| GOU5356 | *tbb-2 (gfp11-i) (syb7618); ujls113; him-5 (e1490);*  *glh-1::t2a::sfgfp1-10 (sam129)* | Genetic cross |
| GOU5057 | *tba-5 (A19V) (qj14)* | Microinjection |
| GOU5058 | *tba-5 (A19V) (qj14); mnIs17; him-5 (e1490)* | Genetic cross |
| GOU5067 | *gfp:tbb-4 (cas2563)* | Microinjection |
| GOU5068 | *tba-5 (A19V) (qj14); cas2563; him-5 (e1490)* | Genetic cross |
| GOU5357 | *tba-5 (A19V) (qj14); mnIs17;*  *Pdyf-1::tba-5 (A19V) (casEx6062)* | Microinjection |
| GOU5358 | *tba-5 (A19V) (qj14);*  *Pdyf-1::tba-5 (A19V) (GFP-N) (casEx6063)* | Microinjection |
| GOU5359 | *tba-5 (A19V) (qj14);*  *Pdyf-1::tba-5 (A19V) (GFP-C) (casEx6064)* | Microinjection |
| GOU5362 | *tba-5 (A19V) (qj14); cas1086;*  *Pdyf-1::qj14 (GFP11-i no linker) (casEx6067)* | Microinjection |
| GOU5363 | *tba-5 (A19V) (qj14); cas1086;*  *Pdyf-1::qj14 (GFP11-i GS-linker 2) (casEx6068)* | Microinjection |
| GOU5364 | *tba-5 (A19V) (qj14); cas1086;*  *Pdyf-1::qj14 (GFP11-i GS-linker 3) (casEx6069)* | Microinjection |
| GOU5365 | *gfp::3xgs::tba-5 (A19V) KI (GFP-N) (cas2560)* | Microinjection |
| GOU5366 | *tba-5 (A19V) (scarlet-i) KI (Scarlet-i) (cas2561)* | Microinjection |
| GOU5367 | *tba-5 (A19V) (gfp11-i) KI (GFP11-i) (cas2562)* | Microinjection |
| GOU5368 | *tba-5 (A19V) (gfp11-i) (cas2562); cas1086* | Genetic cross |
| GOU5369 | *tba-5 (A19V) (gfp-i) (cas2565); cas1086* | Microinjection |
| GOU5377 | *tba-5 (gfp11-i) KI (cas2566); cas1086* | Microinjection |
| GOU1856 | *ebp-2::mneongreen (cas2564)* | Microinjection |
