# Supplementary material for: AlphaFold2-guided engineering of split-GFP technology enables labeling of endogenous tubulins across species while preserving function
Source: PLoS Biol. 2024 Aug 19;22(8):e3002615. doi: 10.1371/journal.pbio.3002615 (PMC11361732; doi:10.1371/journal.pbio.3002615)
Supplement: S2 Table — (DOCX) [file pbio.3002615.s019.docx]

**S2 Table.** CRISPR-Cas9 targets in this study.

| **Gene** | **CRISPR-Cas9 targets (PAM)** | **Application Description** |
| --- | --- | --- |
| *tbb-2* | GAGGTCATCTCCGACGAGCACGG | TBB-2 (GFP11-i) knock-in |
| *tba-5* | CTGTTGGGAACTGTATTGCTTGG | TBA-5 (A19V) knock-in  TBA-5 (GFP-N) knock-in  TBA-5 (GFP11-i) knock-in  TBA-5 (GFP-i) knock-in  TBA-5 (Scarlet-i) knock-in |
