# Supplementary material for: AlphaFold2-guided engineering of split-GFP technology enables labeling of endogenous tubulins across species while preserving function
Source: PLoS Biol. 2024 Aug 19;22(8):e3002615. doi: 10.1371/journal.pbio.3002615 (PMC11361732; doi:10.1371/journal.pbio.3002615)
Supplement: S3 Table — (DOCX) [file pbio.3002615.s020.docx]

**S3 Table. ﻿**Plasmids and Primers in this study.

| **Plasmid** | **Forward Primer** | **Reverse Primer** | **Notes** |
| --- | --- | --- | --- |
| pDONR-P*dyf-1::sfgfp1-10* | TTCTCCTTTGGACATTTTGACAAGCTTACACAGAAATATAGC | GATCCAAATGAAAAATAGGTCCAATTACTCTTCAACATCCCTACA | *sfgfp1-10* fragment was cloned into PCR amplified pDONR backbone using ﻿In-Fusion® HD Cloning Kit |
| pDONR-P*dyf-1::tba-5 (A19V)* | ATCGAAACTATTTCACGCATTTTGACAAGCTTACACAGAA | CAAATGATGAAGAATATTAGGTCCAATTACTCTTCAACAT | *tba-5 (A19V)* cDNA fragment was cloned into PCR amplified pDONR backbone |
| pDONR-P*dyf-1::tba-5 (A19V) (GFP-N)* | CTCCTTTACTCATTTTGACAAGCTTACACAGA | CTGGAAGTGGTAGCGGTGCTGGCAGCGGTAGTGGTATGCGTGAAATAGTTTCGAT | *gfp:3xgs* fragment was cloned into pDONR-P*dyf-1::tba-5 (A19V)* |
| pDONR-P*dyf-1::tba-5 (A19V) (GFP-C)* | CCGCTACCACTTCCAGCTCCACTACCACTTCCAGCATATTCTTCATCATT | GGATGAACTATACAAATAGGTCCAATTACTCTTCAAC | *3xgs::gfp* fragment was cloned into pDONR-P*dyf-1::tba-5 (A19V)* |
| pDONR-P*dyf-1::tba-5 (A19V) (GFP11-i) (no linker)* | CTTCATGAATATGTAAATGCAGCTGGTATAACTGGTGTTGAGGATCAATCG | CTGATGATACAAGCTATGGACGAGATCATATGGTACTTCATGAATATGTAAAT | *gfp11* fragment was cloned into pDONR-P*dyf-1::tba-5 (A19V)* |
| pDONR-P*dyf-1::tba-5 (A19V) (GFP11-i) (GS-linker 2)* | ACTTCCAGCAGTTATACCAGCTGCATTTACATATTCATGAAGTACCATATGATCTCGGCCGCTACCGCTACCG | GGTATAACTGCTGGAAGTGGTAGCGGGGCTGGAAGTGGTAGCGGAGTTGAGGATCAATCGTATAATACC | *GS-linker2::gfp11::GS-linker2* fragment was cloned into pDONR-P*dyf-1::tba-5 (A19V)* |
| pDONR-P*dyf-1::tba-5 (A19V) (GFP11-i) (GS-linker 3)* | ACTTCCAGCAGTTATACCAGCTGCATTTACATATTCATGAAGTACCATATGATCTCGGCCGCTCCCGCTCCCTGC | TATAACTGCTGGAAGTGGTAGCGGGGCTGGAAGTGGTAGCGGAGCGGGCAGTGGATCGGGAGTTGAGGATCAATCGT | *GS-linker3::gfp11::GS-linker3* fragment was cloned into pDONR-P*dyf-1::tba-5 (A19V)* |

| pLV-P*CMV-egfp-htuba1a* | TCGAGCTCAAGCTTCGAATTCGCCACCATGGTGAGCAAGGGCGAGGAGCTGT | TACCCGGTAGAATTATCTAGATTAGTATTCCTCTCCTTCTTCCTCACC | *egfp::linker::htuba1a* cDNA fragment was cloned into PCR amplified pLV backbone |
| --- | --- | --- | --- |
| pLV-P*CMV-htuba1a (gfp11-i)* | TAGCGGGGCTGGAAGTGGTAGCGGAGCGGGCAGTGGAGGAGGAGATGATTCCTTCAACACCTT | CGCCGCTACCGCTACCGGCTCCGCTCCCGCTTCCAGCCCCAATGGTCTTGTCACTTGGCATCT | *GS-linker3::gfp11::GS-linker3* fragment was cloned into pLV-P*CMV-htuba1a* |
| pLV-P*CMV-sfgfp1-10* | CTTTGGACATGGTGCGGCCGCGTGGAT | ATGAAAAATAGGGGCCGCGACTCTAG | *sfgfp1-10* fragment was cloned into PCR amplified pLV backbone |
| pCDNA3.0-P*CMV*-*htuba4a* | CCGCCACCATGCGCGAGTGCATTTCA | GGCCCTCTAGACTACTCTTCTCCCTCATCCTCG | *htuba4a* fragment was cloned into PCR amplified pCDNA3.0 backbone |
| pET27b-*T7-mtuba4a* | CTGAAATGCACTCGCGCATAAGTATATCTCCTTCTTAAAGTTAAACAAAA | CGAGGATGAGGGAGAAGAGTAGGTCGAGTCCAACAAGCTTG | *mtuba4a* cDNA fragment was cloned into PCR amplified pET27b backbone |
| ﻿pDD162-P*eft-*  *3::Cas9* + P*U6::tba-5* sg | TGTTGGGAACTGTATTGCTGTTTTAGAGCTAGAAATAG | CAATACAGTTCCCAACAGCAAGACATCTCGCAATAG | *﻿*PCR from pDD162-P*eft-*  *3::Cas9*+P*U6::Empty* *sgRNA* |
| ﻿pPD95.77-*tba-5 (A19V)* KI genomic  template | CCAAGTGATTAGCAGAGGAGATTAG | ATTCGATCTCATGGGTTCTTTTTCTGTC | *tba-5 (A19V)* genomic fragment was amplified from *qj14* and cloned  into pPD95.77 backbone |
| pPD95.77-*tba-5 (A19V) (GFP-N)* KI template | CCAAATGGAAAAATGAGTAAAGGAGAAGAACTTTTC | GCTGGCAGCGGTAGTGGTATGCGTGAAATAGTTT | *gfp:3xgs* fragment was cloned into pPD95.77-*tba-5 (A19V)* template |
| pPD95.77-*tba-5 (A19V) (Scarlet-i)* KI template | GATGATACAAGCTATGGAGCTGGAAGCGGGAGCGGA | AGCGGAGCAGGTAGTGGTGTTGAGGATCAATCGTAT | *GS-linker3::scarlet::GS-linker3* fragment was cloned into pPD95.77-*tba-5 (A19V)* template |
| pPD95.77-*tba-5 (A19V) (GFP-i)* KI template | TGGAAGTGGGATGAGTAAAGGAGAAGAACTTTTC | CACTACCCGCTTTGTATAGTTCATCCATGCCAT | *gfp* fragment substituted *scarlet* through PCR from pPD95.77-*tba-5 (A19V) (Scarlet-i)* KI template |
| pPD95.77-*tba-5 (A19V) (GFP11-i)* KI template | CAGCTGCATTTACATATTCATGAAGTACCATATGATCTCGCCCACTTCCAGCGC | GAATATGTAAATGCAGCTGGTATAACTGCGGGTAGTGGTAGCGGG | *gfp11* fragment substituted *scarlet* through PCR from pPD95.77-*tba-5 (A19V) (Scarlet-i)* KI template |
