# Supplementary material for: AlphaFold2-guided engineering of split-GFP technology enables labeling of endogenous tubulins across species while preserving function
Source: PLoS Biol. 2024 Aug 19;22(8):e3002615. doi: 10.1371/journal.pbio.3002615 (PMC11361732; doi:10.1371/journal.pbio.3002615)
Supplement: Attachment — Submitted filename: 20240630_Response to reviewers-ou.docx [file pbio.3002615.s022.docx]

June 30^th^, 2024

*PLOS Biology*

Manuscript Number: PBIOLOGY-D-24-00861R1

Authors: Xu et al.

Dear Editor,

Thank you for your letter of June 17^th^, 2024. We have now submitted our revised manuscript entitled “Artificial Intelligence-Enabled AlphaFold II Pipeline Guides Functional Fluorescence Labeling of Tubulin Across Species” to *PLOS Biology.*

We are glad that reviewers and the journal are interested in the topic and our work. As detailed in response to the reviewers, we have carefully addressed each point by additional data presentation and textual clarifications. We appreciate the comments from the reviewers for helping us improve our manuscript.

We thank you for your time and attention and for handling our manuscript.

Sincerely,

Guangshuo

Guangshuo Ou, Ph.D.

Professor

Tsinghua-Peking Center for Life Sciences

School of Life Sciences

Tsinghua University, Beijing 100084, China

E-mail: [guangshuoou@tsinghua.edu.cn](mailto:guangshuoou@tsinghua.edu.cn)

**Point-to-point response for:**

***Artificial Intelligence-Enabled AlphaFold II Pipeline Guides Functional Fluorescence Labeling of Tubulin Across Species.***

*Kaiming Xu et al.*

**RESPONSE TO THE EDITOR**

*********************

Editor Comments:

Thank you for your patience while your manuscript "Artificial Intelligence-Enabled AlphaFold II Pipeline Guides Functional Fluorescence Labeling of Tubulin Across Species" was peer-reviewed at PLOS Biology. It has now been evaluated by the PLOS Biology editors, an Academic Editor with relevant expertise, and by several independent reviewers. I apologize for the delay in coming to this decision.

In light of the reviews, which you will find at the end of this email, we would like to invite you to revise the work to thoroughly address the reviewers' reports.

As you will see below, the reviewers are quite positive, although they do have some concerns and suggestions. The reviewers would like to see more explanation on your developed method, and would like you to include the reasoning behind selecting the specific residue used here. We think that most of the reviewers’ comments are reasonable, although we advise you to not split the manuscript into two (as suggested by R1). Furthermore, while the MAP experiment suggested by R2 and the stable cell line suggested by R3 would undoubtedly strengthen the paper, the Academic Editor considers these to be optional.

Given the extent of revision needed, we cannot make a decision about publication until we have seen the revised manuscript and your response to the reviewers' comments. Your revised manuscript is likely to be sent for further evaluation by all or a subset of the reviewers.

We expect to receive your revised manuscript within 3 months. Please email us (plosbiology@plos.org) if you have any questions or concerns, or would like to request an extension.

At this stage, your manuscript remains formally under active consideration at our journal; please notify us by email if you do not intend to submit a revision so that we may withdraw it.

We thank the Editors for the positive comments on our study and thoughtful assessment of suggestions from the Reviewers. We have now revised our manuscript, and performed all the necessary revisions as indicated below.

**Point-to-point response to the reviewer comments**

*Reviewer #1: This is a very detailed paper with many different experiments, supplemented with computational prediction approaches with AlphaFold II, and fluorescence microscopy.

In essence, this work introduces a amino acid linker system of various length that should facilitate tubulin isotype labeling with GFP, at the inner microtubule lumen. The linker is predicted to avoid clashes with functional MAP interactions in a living cell, an apparent problem with conventical tubulin - GFP tagging. They use human cell lines, murine embryonic tissue, as well as C. elegans as a model system, as it offers many areas of diverse tubulin function in one organism.

We appreciate the Reviewer for the positive assessment of our work, and would like to address all the concerns listed below.

Fig. 1: How unique was the structural prediction shown here? This location seems like a very flexible arrangement. Hence, I would expect a large number of possible solutions.

We provided all PAE (predicted alignment error) plots corresponding to Fig. 1A and Fig. S1E below. We marked all successful binding (GFP11 and GFP1-10) cases by orange circles. These predictions were highly repeatable and reliable using AlphaFold II software. Importantly, all predictions aligned with our live-cell imaging data, as depicted in Fig. S4F (GFP11 without a linker or with a 6-amino acid GS-linker 1 showed no detectable GFP signal, whereas those with 12- or 16-amino acid linkers emitted fluorescence). Therefore, the structural predictions in Fig. 1 are unique.

We have now provided the information in the revised text:

(Page 8, line 184) ‘These predictions were consistent with our live-cell imaging results (Fig. S4 F), confirming the uniqueness and reliability of our structural predictions…’

Fig. 2: What is the ratio between of tagged tubulin and endogenous tubulin in each case tested? Was the tagged tubulin the only available variant, or was this a mix with untagged tubulin?

In Fig. 2, we utilized genome editing technology to create homozygous *gfp11-i* tagged endogenous β-tubulin *tbb-2* animals through knock-in. Consequently, all endogenous TBB-2 tubulins were labeled with GFP11-i tags. Previous studies have demonstrated that, alongside TBB-2, TBB-1 is also highly expressed during early-stage embryonic development in C. elegans (Honda et al., 2017; Lu and Zheng, 2022). We have incorporated this information into the revised text:

(Page 9, line 199) ‘…Utilizing CRISPR-Cas9 genome editing technology (Cong et al., 2013), we integrated either GFP or GFP11-i into the C. elegans genome, resulting in all endogenous TBB-2 being tagged.’

(Page 9, line 210) ‘As the embryos of *C. elegans* contain a mixture of tagged TBB-2 and untagged TBB-1, …’

The link to Tubulinopathies is a far distance claim. Obviously, every useful method might add some knowledge to diseases and pathological conditions, but this paper is a structural study into a method improvement (GFP tagging of tubulin) that may, or may not contribute anything.

We agree with the Reviewer that the development of a method may not necessarily lead to discoveries if the method does not solve a problem. In contrast, our study addresses a significant gap in existing methods. A primary motivation for developing this method is the limitation of current approaches in faithfully tracking the dynamics of pathogenic tubulin in living organisms or cells. As described in this work, pathogenic tubulin mutations lose their toxic effects on MTs when labeled with GFP at either the N- or C- terminus, which hinders mechanistic studies of their cellular effects. With our developed method, we can observe the dynamic behavior of these pathogenic tubulins while preserving their ‘toxicity’ on MTs, promising to advance our mechanistic understanding of their cellular impact. We have incorporated this discussion into the revised text:

(Page 19, line 484) ‘Considering that pathogenic tubulins lose their toxic effects on MTs when labeled with GFP (Fig. 6), our developed method enables following the dynamic behavior of pathogenic tubulins in living organisms or cells while preserving their ‘toxicity’ on MTs, thereby holding the promise to advance mechanistic understanding of ciliopathies and other tubulin-related diseases involving tubulin isotypes. (Cushion et al., 2023; Tischfield et al., 2011).’

I am not so convinced that the linker between GFP and tubulin is really necessary to maintain function. GFP tagging has been widely used in the past with no obvious interferences of GFP and tubulin function, mainly because only a fraction of tagged protein is necessary to achieve a good fluorescent signal. Hence, leaving large areas of microtubules free for MAP interactions, inside the barrel, or outside. However, I do not want to diminish the value of this work, but I am confused by the presentation and the lack of focus.

Indeed, the overexpression of GFP-tagged wild-type tubulin has been widely employed to monitor the dynamic behavior of tubulin and MTs. As noted by the Reviewer, a small fraction of GFP-tagged protein yields a robust fluorescent signal. However, this field has recognized that tagging tubulin with GFP at either the N- or C-terminus can compromise its functionality when employing knock-in strategies to label endogenous tubulins. Instead, our approach involves inserting the GFP11 tag into the H1-S2 loop of tubulin, along with a 16-amino acid linker sequence. This method not only preserves the functionality of endogenous wild-type tubulin but also retains the cellular effects of pathogenic tubulin. We have elaborated on this issue in the revised Introduction section:

(Page 5, line 109) ‘…ectopic over-expression of wild-type GFP-tagged tubulin isotypes has been widely adopted across diverse cellular environments, including the nematode (Hao et al., 2011), cell lines (Mora-Bermudez et al., 2007) and mouse embryos (Na and Zernicka-Goetz, 2006), with a fraction of GFP-tagged tubulins providing strong fluorescent signals.’

My recommendation to the authors for this paper is, focusing on the methods, and their validation with the necessary experiments (further emphasizing examples where the conventional tagging indeed caused a problem like in embryos), and argue more convincingly about the usefulness of structure prediction software (AlphaFold II in this case for the design of the linker). It seems to me that this paper could easily being split into two papers, a method focused one and one with a focus on the actual biology that is shown here. Currently, this paper is too long, too complex, and therefore hard to read.

I will not say that the data is bad, but the presentation is confusing with over-stuffed figures, and with no strong focus on the apparent outreaching advantages of the methods shown here. This paper could be very interesting to a larger community, but it should be revised to a form that addresses a broad scientific community.

We appreciate the Reviewer’s overall positive comments and have consolidated the manuscript into one as suggested by the Editor. Moreover, we have made extensive efforts to clarify many issues to enhance readability for a wider scientific audience.

*Reviewer #2: The manuscript entitled "Artificial Intelligence-Enabled AlphaFold II Pipeline Guides Functional Fluorescence Labeling of Tubulin Across Species" elegantly addresses a major challenge in the tubulin field: correctly tagging alpha and beta tubulin isoforms. To date, most tags used to visualise tubulin are placed at the C-terminal or N-terminal region of the protein, which negatively affects its dynamics and proper protein-protein recognition and interaction. To tackle this issue, Kaiming Xu et al. argue that regions within the protein sequence with low conservation are the best option for tagging. They selected the H1-S2 loop in tubulin and began searching for the best option for fluorescently labeling this protein. They performed Rosetta relax runs to determine how the fluorescent protein would affect the folding of tubulin. They discovered that full-length versions of fluorescent proteins negatively impact tubulin folding. Consequently, they decided to use a split version of GFP (GFP11).

Next, they searched for the optimal linker length to attach GFP11 to the H1-S2 loop using AlphaFold II, discovering that, of the three linkers they tested, the best option was the 16 aa (GS) linker. Following this optimization, they demonstrated their improved tubulin tagging method in different models, including both alpha and beta isoforms. They conducted impressive work in C. elegans, showing that this tagging does not affect tubulin properties, unlike current N-terminal and C-terminal tags. They tested overexpression as well as endogenous tagging of different tubulin isotypes and mutated versions, showing that tubulin function remains unaffected in various scenarios. Finally, they applied this method to mammalian tubules from different sources, from human tumor cells to mouse oocytes, demonstrating that this tagging approach is useful for studying the tubulin code in both physiological and pathological environments. Notably, in addition to not affecting tubulin properties, this tagging method provides a higher signal-to-noise ratio, which is very useful in cell work.

In my opinion, the current manuscript represents a significant advancement in protein tagging, particularly for tubulin fluorescent labelling, and it can be a substantial improvement in the study of the tubulin code, focusing on tubulin isotype composition and function in cells. I believe the manuscript should be accepted with minor revisions. Please find some comments and suggestions below:

We appreciate the Reviewer for the supportive comments on our work.

(i) I would appreciate a basic schematic of the pipeline used to design and improve the tagging in Figure 1. This would greatly aid in understanding the bioinformatics aspect of this work.

We thank the Reviewer for this suggestion. Now we have provided this schematic of the pipeline as ‘Fig. S1 F’ in the revised manuscript:

(Page 9, line 194) ‘… A schematic of AlphaFold II pipeline-guided design of functional protein labeling was shown in Fig. S1 F.’

(Page 46, line 1034) ‘…(F) Schematic of AlphaFold II (AF2)-guided design of functional tagging of tubulins or other proteins (Protein A). MSA, multiple sequence alignment…’

(ii) The authors directly label the H1-S2 loop, although they mention that low-conservation regions within the sequence could be good options for tagging. Have they explored other regions? It would be a good control of their pipeline to compare other options with the chosen one, at least in silico.

We initially chose to label the H1-S2 loop directly based on early genetic and biochemical findings. Schatz et al. (1987) and other studies demonstrated that inserting short sequences or small affinity tags into the H1-S2 loop of α-tubulins did not disrupt their function (Page 7, line 141). In the context of tubulin structure within MTs (Fig. 1 B, middle panel), the H1-S2 loop (inner lumen surface) and the C-terminal tail (outer surface) are the only low-conserved regions available for GFP11 insertion, thereby exposing the GFP11 tag to the MT surface and facilitating further binding with GFP1-10. Tagging proteins to the C-terminal tail disturbs tubulin function and interaction with microtubule-associated proteins (MAPs) including motor proteins. Therefore, the H1-S2 loop emerged as the sole candidate for functional labeling of both α- and β-tubulins. However, we also acknowledge the possibility of other regions suitable for labeling.

(iii) Additionally, I miss some in vitro experiments or at least some in silico designs of other tags besides fluorescent proteins. Have the authors considered other tags, such as those used for purification?

Schatz et al. (1987) and subsequent studies have successfully inserted 6xHis affinity tags into the H1-S2 loop of α-tubulin for purifying tubulins from yeast. Hence, we acknowledge that inserting other small tags would indeed be feasible. The innovation of our approach lies in functional labeling of tubulin isotypes within live-cell systems, thereby enabling the study of tubulin dynamics in both physiological and pathological contexts. We have discussed this issue in the revised text:

(Page 18, line 463) ‘…Therefore, inserting other small tags into the H1-S2 loop would be also feasible for tubulin purification….’

(iv) As mentioned in the discussion section, it would be helpful to demonstrate that MAPs binding is not affected by this tagging (they focus on luminal MAPs, but I would check others as well). Have the authors considered purifying and injecting some fluorescent-MAPs to study this?

We agree with the Reviewer for this excellent suggestion. We measured the velocity of kinesins (MAPs binding to the outer surface of MTs) along MTs in nematode cilia. Using the intraflagellar transport component DYF-11 as markers, we found that the velocity of ciliary kinesins in GFP11-i tagged TBA-5 animals was indistinguishable from that of wild-type animals (Fig. S5 B and C), suggesting that GFP11-i labeling may not disrupt interaction of MTs with other MAPs. However, we can not rule out its potential effects on other MAPs. We have addressed this issue in the revised text:

(Page 12, line 304) ‘…Using the intraflagellar transport component DYF-11 as markers, we found the velocity of ciliary kinesins in GFP11-i tagged TBA-5 animals was also indistinguishable from that of wild-type animals (Fig. S5 B and C), implying that GFP11-i labeling may not disturb interaction between MTs and MAPs.’

(v) The units used to measure brood size are missing from the text and Figures 2C and 2D.

We have now refined the textual clarifications in the figure legends of Fig. 2 C and 2 D in the revised manuscript. We thank the Reviewer for point out this issue:

(Page 36, line 924) ‘The number indicated amounts of hatch worms.’

*Reviewer #3: The manuscript by Kaiming Xu and colleagues from the laboratory of Guangshuo Ou reports a novel fluorescent labelling strategy of tubulin that improves its polymerization capacities and limits its interference with the co-assembly with other non-labelled tubulins. The strategy is based on numerical simulations of the conformation of tubulins, which take advantage of artificial intelligence to identify the residues to which the labelling would not interfere with protofilament elongation and microtubule assembly. The authors demonstrate that the direct binding of GFP to these residues is incompatible with microtubule elongation. However, they found that the binding of a portion of GFP to the tubulin in parallel with the co-expression of the complementary portion in the cytoplasm is fully functional. The co-expression of the two constructs in the C. elegans embryo led to the formation of dynamic microtubules, which exhibited an improved signal-to-noise ratio when imaged compared to the results obtained using classical strategies.

Subsequently, the authors demonstrated that the expression of specific isotypes of tubulin in a defined lineage of the worm enables the assembly of fully functional microtubules in the cell body and their cilia. Moreover, the same strategy could be applied to mammalian cells in culture and to mouse embryos. In conclusion, these data are highly promising and compelling, and the presented strategy is likely to be of broad interest, initially in the field of tubulins and microtubules, but also in other areas such as non-perturbing labelling of proteins in living cells and organisms.

We appreciate the Reviewer for the supportive comments on our work and constructive suggestions.

There is already a large amount of data in this manuscript which can be published as it is and I don't want to ask more to the authors. I have no expertise in the use of artificial intelligence and don't know much about ciliogenesis in C elegans. However I have some experience in the study of microtubules in mamalian cells in culture. And I think the author are in position to overcome a great limitation in the field which is the generation of stable cell lines expressing a non-perturbing form of fluorescently labelled tubulin. Probably for the reasons of poisoning discussed in the introduction of the manuscript, there is currently no such cell lines. Colleagues have to work on transient transfections or microinjection of tubulin. To my knowledge, no lab has generated a stable cell line in which microtubules would have a proper dynamic. Such a stable cell line would be of great use to the community and would significantly improve the impact of the work. Since the work has been done in HeLa I guess authors could generate a stable line from HeLa but a cell line with a proper control of mitosis and with good ciliogenesis potential would be much more useful. Hence I recommend the author to generate a cell line expressing their two constructs from RPE1 (a non-transformed human cell line often used to study mitosis and ciliogenesis).

We agree that constructing a stable cell line expressing fluorescently labeled tubulins would be valuable for the community and would enhance the impact of this work. However, creating the correct knock-in cell line will require several months of effort. As suggested by the Editor, we propose to defer this experiment for future research to ensure timely publication of the current study. We have outlined this plan in the revised manuscript:

(Page 19, line 506) ‘...Future studies are required to explore generating cell lines or mouse models expressing GFP11-i labeled tubulins to investigate MT dynamics in various MT-based processes, such as mitosis or ciliogenesis.....’
